# Disease and Economic Burden Averted by Hib Vaccination in 160 Countries: A Machine-Learning Analysis

**DOI:** 10.3390/vaccines13121197

**Published:** 2025-11-27

**Authors:** Dachuang Zhou, Siyang Chan, Yimei Zhong, Zhehong Xu, Jun Wang, Yuntian Wang, Yiyang Gao, Yuting Xia, Di Zhang, Wenxi Tang

**Affiliations:** 1 Center for Pharmacoeconomics and Outcomes Research, China Pharmaceutical University, Nanjing 211198, China; 3122044202@stu.cpu.edu.cn (D.Z.); 3224041316@stu.cpu.edu.cn (S.C.); 3323041389@stu.cpu.edu.cn (Y.Z.); 3324041403@stu.cpu.edu.cn (Y.G.); xyt_shama@stu.cpu.edu.cn (Y.X.); z2113941536@163.com (D.Z.); 2 Department of Public Affairs Management, School of International Pharmaceutical Business, China Pharmaceutical University, 639#Longmian Road, Nanjing 211198, China; 3 Paula and Gregory Chow Institute for Studies in Economics, Xiamen University, Xiamen 361005, China; zhehongxu@stu.xmu.edu.cn; 4 State Key Laboratory of Drug Research, Molecular Imaging Center, Shanghai Institute of Materia Medica, Chinese Academy of Sciences, Shanghai 201203, China; wangjun1@simm.ac.cn; 5 The State Key Laboratory of Multimodal Artificial Intelligence Systems, Institute of Automation, Chinese Academy of Sciences, Beijing 100190, China; 22120854000025@hainanu.edu.cn; 6 The School of Information and Communication Engineering, Hainan University, Haikou 570228, China

**Keywords:** Haemophilus influenzae type b, cost-effectiveness, vaccination

## Abstract

Background: Global immunization against Haemophilus influenzae type b (Hib) has expanded with Gavi support. We estimated health, economic benefits, equity and cost-effectiveness in 159 countries (1990–2021), and projected effects of future introduction in China. Methods: We used a random forest model to simulate counterfactual scenarios without Hib vaccine introduction in 159 countries (1990–2021) and to project effects of Hib vaccine introduction in China over the next decade. Ten variables were sourced from the World Bank and WHO; Hib disease burden estimates were from the Global Burden of Disease Study 2021. We compared counterfactual and actual results to quantify benefits, equity, and cost-effectiveness. Extensive uncertainty analyses were performed. Results: Between 1990 and 2021, Hib immunization averted an estimated 1,321,123 (95% uncertainty interval [UI] 32,034–2,723,304) deaths and 90,973,504 (95% UI 3,573,718–197,099,799) disability-adjusted life-years globally. Greatest health and economic gains occurred in Africa and low- and middle-income countries (LMICs). Deaths averted decreased with later vaccine introduction (Pearson’s r = −0.56). Vaccination did not improve health equity, and access remains limited in Africa and LMICs. Hib immunization was cost-saving in all countries. In China, introduction at any point in the next decade would provide health and economic benefits and be cost-effective, with earlier introduction yielding greater gains. Conclusions: Hib immunization provide substantial, cost-effective health and economic benefits globally. Persistent inequities in vaccine access for LMICs require targeted solutions. Policymakers in China should consider these findings for future vaccine introduction.

## 1. Introduction

Haemophilus influenzae type b (Hib) is a major cause of severe diseases such as pneumonia, meningitis, and sepsis in children [1,2,3]. Hib vaccination is the only proven public health intervention to effectively reduce Hib-related morbidity and mortality [4,5,6]. Since its introduction in 1987, Hib vaccine has led to near-elimination of Hib disease in early-adopting high-income countries [7,8,9].

At the beginning of this century, high vaccine prices limited Hib vaccine access in low- and middle-income countries (LMICs), where most Hib-related deaths occurred [10,11]. In response, Gavi, the Vaccine Alliance, initiated a global effort in 2005, reducing vaccine prices and accelerating introduction [12]. As a result, nearly all countries now include Hib vaccine in national programs [12]. Substantial declines in Hib disease burden have been documented in many low-income countries [13,14,15]. Yet, as of 2021, 88.3% of Hib-related deaths still occurred in LMICs, underscoring inequities in vaccine access [11].

Although Hib disease burden has dropped following vaccine introduction, some studies caution that attributing all declines to vaccination may overestimate its impact, as health systems and medical care have also improved [16,17]. Our comprehensive search identified scattered country-level studies reporting reductions in disease burden following vaccine introduction (Tables S1 and S2). However, these evaluations were limited in scope, covering only a small number of countries, and no global or systematic assessment has yet been conducted. Moreover, there remains a lack of comprehensive analyses on the economic value of Hib immunization, including human capital losses due to premature mortality. Although several studies from high-income countries have suggested that Hib vaccines are highly cost-effective (Table S2), evidence from low- and middle-income countries is insufficient. In addition, China is the only country yet to include Hib vaccine in its national immunization schedule. While the vaccine is available for private purchase, low awareness and high out-of-pocket costs have led to coverage below 5% [18]. Despite repeated calls for introduction in China, research on the optimal timing to maximize health and economic benefits is limited [19,20,21].

Key scientific questions remain unanswered and are critical for optimizing resource allocation and policy decisions regarding Hib vaccination: (1) What are the overall health benefits of Hib immunization at global, regional, and income-level strata? (2) Has global Hib vaccination improved health equity? (3) Is continued Hib vaccination necessary in high-income countries where disease burden is now minimal? (4) What are the economic gains from Hib vaccination in terms of mortality averted, and does vaccine procurement represent good value for money? (5) For China, what is the optimal timeline for Hib vaccine introduction to maximize health and economic returns? Addressing these questions is essential for evaluating past policies and guiding future public health strategies.

This study advances previous research comprehensively in terms of methodology (machine learning-based counterfactual simulation, excluding confounding factors such as economic development and improvements in medical care), scale (analysis covering 160 countries, with subgroup analyses by continent and income level), and outcomes (health benefits, economic gains, health equity index, cost-effectiveness, and correlation analyses). This study provides clear evidence to directly inform national and global immunization policy decisions and to support strategic planning for future vaccine programs. In this study, we analyze data from 159 countries (1990–2021) using machine learning-based counterfactual models to estimate the burden that would have occurred without Hib vaccine, quantify health gains, assess the necessity of continued vaccination in high-income countries, examine impacts on health equity, compute economic benefits and cost-effectiveness, and project the impact of Hib vaccine introduction in China.

## 2. Methods

### 2.1. Study Design and Data Sources

We used a machine learning-based causal inference approach to estimate the counterfactual Hib-related disease burden (deaths and Disability adjusted of life years [DALYs]) in the absence of vaccine introduction, and quantified the health benefits by comparing these estimates with observed data. Subgroup analyses by continent, income level, and Gavi support, as well as assessment of health equity and timing of vaccine introduction, were conducted. Economic benefits and cost-effectiveness were estimated using avoided human capital losses and vaccine procurement costs, respectively, with detailed methodology provided in the Figure S1.

Key variables required were sourced from the World Bank, WHO, and the Global Burden of Disease Study 2021 (GBD 2021) [11,22]. Data on vaccine doses and prices were derived from WHO reports [23]. Additional inputs (e.g., labor participation rates, GDP, labor income share) were drawn from the International Labor Organization and Penn World Table [11,24,25,26]. Considering that many countries have consecutive missing values for certain variables, when linear interpolation and similar methods are not feasible, we use per capita GDP—which has very few missing values—as a reference to impute other variables (Tables S3–S5) [27,28].

### 2.2. Counterfactual Simulation Using Machine Learning

We employed a causal forest machine learning model to estimate the counterfactual Hib disease burden in the absence of vaccine introduction [29,30], using covariates including demographic, economic, health system, and vaccine-related factors. Model outcomes included Hib-related deaths, DALYs, and vaccine doses. Countries were analyzed as clustered units. 

### 2.3. Health Benefits Analysis of Hib Immunization

Annual health benefits were estimated as the difference between observed and counterfactual values for each country, with global and subgroup summaries by continent, income, and Gavi status. Health equity was assessed using the Gini coefficient and Theil index for disease burden in 1990 and 2021. 

### 2.4. Economic Benefits Analysis of Hib Immunization

Economic benefits were assessed using the human capital approach, combining deaths averted with country-specific GDP and labor force data [31,32]. All benefits were discounted to 2021 at 5% [33]. The economic benefit-to-GDP per capita ratio was also reported.

### 2.5. Cost-Effectiveness Analysis of Hib Immunization

Cost-effectiveness was evaluated by comparing incremental vaccine doses, costs, and DALYs between scenarios. Incremental cost-effectiveness ratio (ICER) was calculated and assessed based on WHO-recommended thresholds, with all costs discounted to 2021 [33,34].

### 2.6. China-Specific Scenario Projection

For China, we projected Hib disease burden, economic benefits, and cost-effectiveness under nine introduction scenarios (2026–2033 and no introduction) using an autoregressive integrated moving average (ARIMA) model and machine learning [35]. All values were discounted to 2021. 

### 2.7. Uncertainty Analysis

We conducted extensive sensitivity analyses. For the counterfactual scenarios simulated using machine learning, we report the lower and upper bounds of the confidence intervals for estimated health benefits. Given parameter uncertainty in the economic and cost-effectiveness analyses, we performed 10,000 Monte Carlo simulations, allowing all parameters to vary up to 10% above their point estimates, and report the lower and upper bounds of the resulting confidence intervals [36]. In addition, due to the uncertainty surrounding the discount rate, we further considered scenarios with a 3% discount rate and no discounting, in comparison with the base scenario, to assess the robustness of the results. As the current Hib vaccine price in China is substantially higher than the global average, and to account for potential price reductions following inclusion in the national immunization, we also report cost-effectiveness results for China assuming a 50% reduction in vaccine price and using the global median vaccine price (USD 0.86) [23].

## 3. Results

### 3.1. Health Benefits Analysis of Hib Vaccination

The machine learning causal forest model demonstrated high predictive reliability, as indicated by root mean square error values ranging from 0.11 to 0.12 [37]. The relative importance of all control variables is provided in the appendix (Table S6).

**Figure 1:**
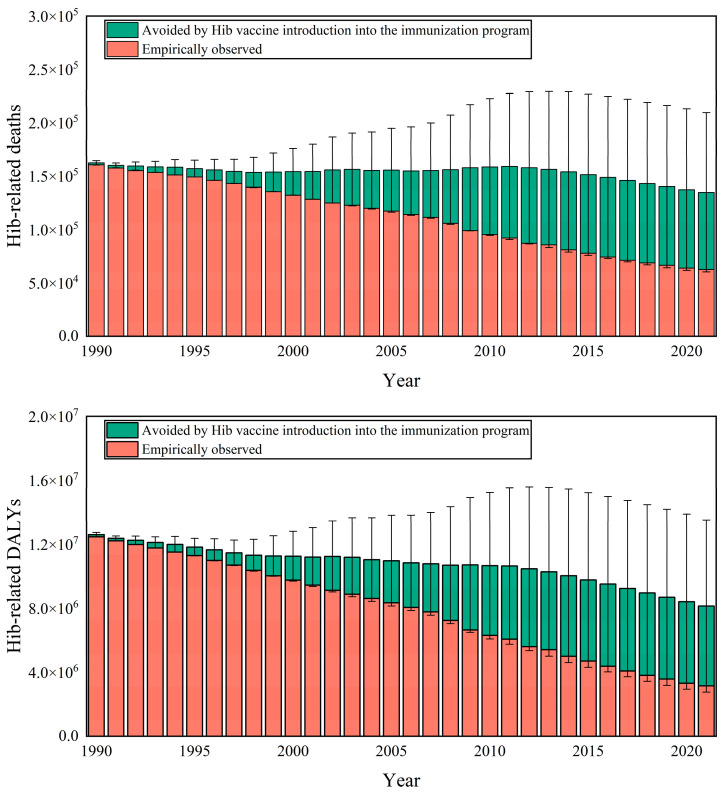
Global Health benefits of Hib immunization in 1990–2021.

As shown in Figure 1, from 1990 to 2021, the global Hib immunization is estimated to have averted 1,321,123 (32,034–2,723,304) deaths (26.88% [0.65–55.42%]) and 90,973,504 (3,573,718–197,099,799) DALYs (26.49% [1.04–57.39%]). Both mortality and DALY rates declined substantially during this period (Figure S3). The annually averted disease burden increased over time with more countries introducing Hib vaccine and sustained coverage leading to herd immunity.

As shown in Table 1, at the regional level, Europe and high-income countries introduced Hib vaccine earliest, with a median inclusion time exceeding 20 years. More than 50% of countries in these groups had introduced Hib vaccine by 1994 (Europe) and 1998 (high-income). In contrast, this threshold was reached in Africa (2008), Asia (2009), and low-income countries (2008) much later. Later introduction, but relatively higher baseline disease burden and relatively weaker healthcare systems, make Africa and low-income countries present a ‘latecomer high-return’ pattern: each vaccination unit brings greater reductions in deaths and DALYs avoided. Cumulative health benefits were higher in African and low-income countries than in other subgroups (Figures S4–S15).

**Figure 2:**
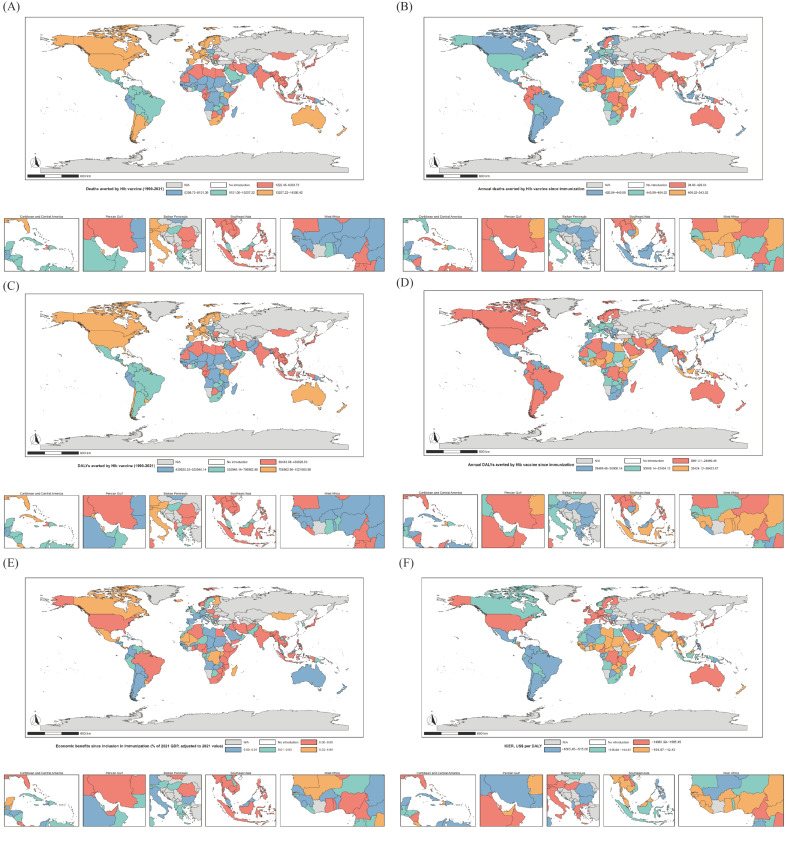
Country-level health, economic, and cost-effectiveness benefits of Hib immunization, 1990–2021. (**A**): Total deaths averted by Hib vaccine; (**B**): Annual deaths averted by Hib vaccine since immunization; (**C**): Total DALYs averted by Hib vaccine; (**D**): Annual DALYs averted by Hib vaccine since immunization; (**E**): Economic benefits of Hib immunization; (**F**): Cost-effectiveness analysis of Hib vaccination.

As shown in Figure 2, high-income countries, despite having a lower baseline disease burden, benefit from early vaccine introduction, high coverage, and well-established vaccination systems, leading to a larger absolute reduction in deaths. In contrast, several African countries, characterized by higher baseline disease burdens and rapid expansion of vaccine coverage post-introduction, exhibit a steeper annual decline in mortality. At the national level, largest numbers of deaths averted by Hib vaccination were observed in Switzerland, Germany, and the United States of America, while the fewest were recorded in Thailand, Iran, and Egypt. The greatest average annual reductions in mortality following the introduction of Hib vaccination were seen in Comoros, Rwanda, and Burundi, whereas Thailand, the Republic of Korea, and Cameroon had the smallest annual declines (Table S7). These findings provide a foundation for optimizing future targeted strategies, such as focusing on high-risk regions and populations.

### 3.2. Health Equity Assessment

**Figure 3:**
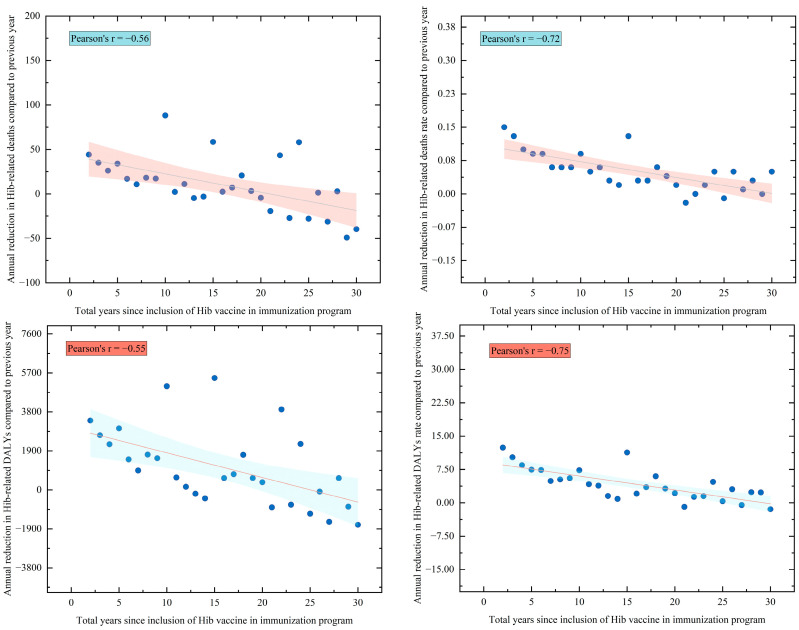
Correlation between health benefits and year of Hib vaccine introduction. Note: Each point represents the mean value for all countries that meet the criteria, and the line is the Pearson fit curve.

Despite widespread Hib vaccine implementation, health inequalities were not alleviated (Tables S9, S10 and Figure S18). Between 1990 and 2021, there was little change in the relative burden of Hib disease across countries, and indices of health equity remained largely unchanged. Notably, the Gini coefficient for the counterfactual scenario in 2021 was lower than for the observed scenario (0.36 vs. 0.80), while the concentration index was also lower (−0.07 vs. −0.15). Furthermore, as shown in Figure 3, we observed diminishing marginal benefits from continued vaccine introduction (Pearson’s r < −0.50). Our analysis, combining Gini and concentration indices, suggests that Hib vaccine introduction between 1990 and 2021 did not resolve health inequity, and may have exacerbated disparities due to unequal vaccine access, with the disease burden remaining concentrated in low- and middle-income countries.

### 3.3. Economic Benefits and Cost-Effectiveness Analysis of Hib Vaccination

As shown in Figure 2, Hib vaccination can prevent a substantial number of premature deaths among children, thereby generating considerable health gains and averting significant productivity losses. Therefore, all countries accrued some degree of economic benefit from Hib immunization, with the greatest proportional benefits observed in African and low- and middle-income countries. Expressed as a percentage of GDP in 2021, the largest economic benefits were seen in San Marino (493.85% [416.61–623.43]), Tuvalu (370.96% [320.18–440.17]), and Palau (370.95% [315.37–509.62]), while India (0.00% [0.00–0.00]), Indonesia (0.01% [0.01–0.02]), and the United Republic of Tanzania (0.01% [0.01–0.02]) experienced the least. Despite lower vaccine prices in Africa and low- and middle-income countries (Figure S2), additional procurement costs as a share of GDP were substantially higher in these regions (Figure S20). Cost-effectiveness analyses indicated that Hib immunization was cost-saving in all countries, with the most favorable ICERs observed in Monaco (−14,981.99 [−18,738.46 to −12,776.83]), Qatar (−9855.54 [−10,946.08 to −8973.10]), and Singapore (−9516.98 [−9779.89 to −8867.99]) (Table S11). The Monte Carlo simulations demonstrate stable results, including under lower discount rates (no discounting or a 3% rate). Accordingly, for high-income countries where Hib vaccination has long been implemented, the ICER confirms the appropriateness of this health policy decision from both health and economic perspectives. Even in countries with low vaccine prices but constrained fiscal space, mechanisms such as Gavi support can enable the realization of long-term cost savings without increasing short-term fiscal pressures.

### 3.4. Results of China-Specific Scenario Projection

**Figure 4:**
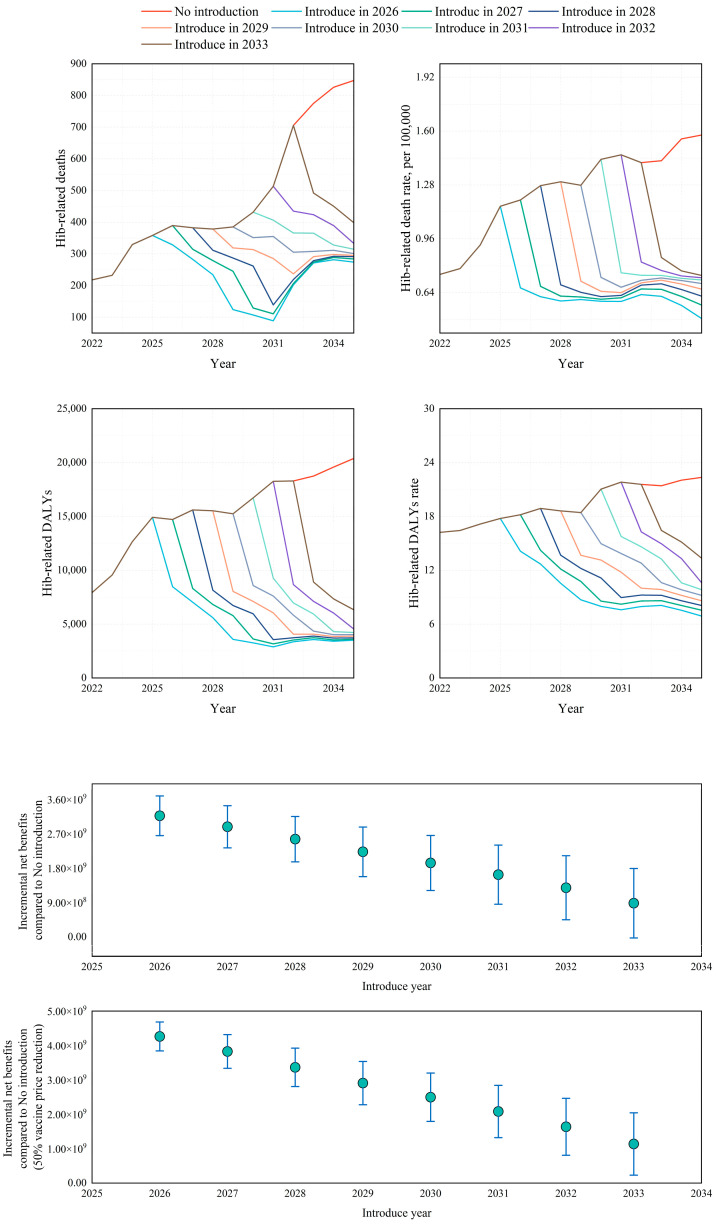
Projected health and economic impact of Hib immunization introduction in China, 2026–2035.

As shown in Figure 4, China, the only country yet to include Hib vaccination in its national immunization, was projected to achieve substantial health and economic gains and cost-effectiveness under any scenario of Hib vaccine introduction between 2026 and 2033. Earlier inclusion was associated with greater benefits. This is partly because earlier adoption facilitates the rapid achievement of high vaccination coverage and herd immunity, generating health benefits that extend well beyond vaccinated individuals. In addition, earlier inclusion carries greater time value when assessed from the present perspective. Compared with no introduction, inclusion in 2026 was projected to avert 3440 (392–4759) deaths and 128,386.95 (36,021.01–306,686.77) DALYs over the subsequent decade, while introduction in 2033 would avert 1108 (161–3017) deaths and 36,139.21 (10,357.60–95,461.77) DALYs. The incremental net benefits compared with no introduction ranged from USD 88 million (2033 introduction [–22 to 200]) to USD 317 million (2026 introduction [254–381]). If vaccine prices declined by 50%, the incremental net benefit increased to USD 114 million (4–226) to USD 426 million (375–478). Using the median global vaccine price, all scenarios were cost-saving.

## 4. Discussion

Over the past three decades, with the support of Gavi and other international partners, nearly all countries—except China—have incorporated Hib vaccine into their national immunization [28]. While several studies have reported reductions in disease burden following introduction, attributing all these health gains solely to immunization may overstate the vaccine’s contribution [16,17]. This study advances previous research comprehensively in terms of methodology (machine learning-based counterfactual simulation, excluding confounding factors such as economic development and improvements in medical care), scale (analysis covering 160 countries, with subgroup analyses by continent and income level), and outputs (health benefits, economic gains, cost-effectiveness, health equity index, and correlation analyses). This study provides clear evidence to directly inform national and global immunization policy decisions and to support strategic planning for future vaccine programs.

Our findings underscore substantial health and economic gains, as well as cost-savings, associated with Hib immunization in diverse settings. However, we also observed persistent and, in some cases, exacerbated inequities in health outcomes, driven in part by disparities in vaccine access [19,38,39,40]. These results highlight the dual need to sustain immunization efforts in countries where benefits remain high, and to strengthen international support for countries facing barriers to Hib vaccine access. Furthermore, our projections for China provide robust evidence supporting prompt inclusion of Hib vaccine in the national immunization schedule.

Driven by sustained advocacy from Gavi and other agencies, middle- and low-income countries progressively adopted Hib vaccine following its earlier uptake by high-income countries. Between 1990 and 2021, Hib vaccination was estimated to have averted 1,321,123 (32,034–2,723,304) deaths and 90,973,504 (3,573,718–197,099,799) DALYs globally, corresponding to reductions of 26.88% (0.65–55.42) and 26.49% (1.04–57.39), respectively. Despite earlier and longer-standing implementation in high-income countries, the greatest absolute health benefits accrued in Africa, Asia, and low-income countries, reflecting both higher baseline disease burden and weaker health system infrastructure [41,42,43]. These findings attest to the critical importance of sustained international financing and technical support in maximizing vaccine impact in settings of greatest need [44,45].

Despite the remarkable global progress, our counterfactual analyses indicated that health inequities have not been reduced and may in fact have worsened. This is consistent with previous findings on childhood vaccination coverage, which indicate that in middle- and low-income countries, relatively weak health systems, fragile supply chains, and limited vaccine management capacity make it more prone to vaccine shortages [46,47,48]. Despite the likelihood that these countries have included vaccines in their immunization programs, children in these nations are at a higher risk than those in high-income countries of failing to complete their full vaccination schedules on time. In 2021, the Gini coefficient and concentration index for Hib disease burden were higher under observed conditions than under a no-vaccine scenario (Gini 0.80 vs. 0.36; concentration index −0.15 vs. −0.07), consistent with previous reports of irregular vaccine supply and unequal access in some low-income countries [19,38,39,40]. Our analysis further revealed diminishing marginal health gains with increasing time since vaccine introduction (Pearson’s r < −0.50), suggesting that, while herd immunity has been achieved in many high-income settings, the greatest potential for health improvement now lies in countries with delayed or incomplete vaccine access. Although Gavi’s efforts have led to substantial reductions in vaccine price for low- and middle-income countries, the proportional economic burden of vaccine procurement remains higher in these settings due to weaker economic capacity. Our findings support continued international investment to ensure stable vaccine supply, strengthen logistics, and target support to vulnerable and marginalized populations to reduce the risk of a “vaccine gap” driven by geographic or economic factors [44,45].

Contrary to the notion that “vaccines are primarily an issue for developing countries” [49,50], our study demonstrates that Hib immunization delivers meaningful economic benefits and cost-savings in all country contexts. While countries with higher baseline disease burden realize greater health improvements, high-income countries benefit from the protection of substantial human capital, with averted childhood deaths translating into significant economic gains [51,52]. Importantly, our cost-effectiveness analysis showed that Hib immunization is cost-saving across all settings, reinforcing the view that vaccines are a global health priority, not only a concern for low- and middle-income countries.

For China, our projections indicate that early introduction of Hib vaccine into the national immunization would yield substantial health and economic gains and be highly cost-effective, despite the current vaccine price (USD 11.62) being much higher than the global median (USD 0.86). Even with a 50% price reduction, cost-effectiveness would be maintained, and price negotiation or pooled procurement could further enhance affordability [53,54]. As more combination vaccines become available and market competition increases, manufacturers are likely to lower prices to maintain market share. Pilot implementation in provinces with high cost-effectiveness could offer a scalable pathway for national adoption [55].

This study has several limitations. First, key input data were sourced from global databases (GBD, World Bank, WHO), which, despite their authority, may contain discrepancies in data collection and modelling approaches, potentially introducing information bias or error. Second, missing data for certain countries and years were addressed using imputation and extrapolation, but these methods may introduce systematic error and affect the accuracy of counterfactual predictions. Third, our use of causal forest models to estimate counterfactual scenarios relies on assumptions of comparability and ignorability, which may be violated in the presence of unmeasured confounders such as health system efficiency or vaccine acceptance. The use of country-level aggregates rather than individual-level data may also introduce ecological bias. Furthermore, this approach may result in relatively wide uncertainty intervals. Economic benefit estimates applied the human capital approach, assumed all Hib-related deaths occurred at age one, and did not discount future productivity losses, potentially underestimating true benefits. We also did not include long-term sequelae among survivors. The use of a uniform 5% discount rate, though standard, may not reflect social preferences across countries. Finally, projections for China were based on quantile regression forests and ARIMA models optimized for minimal RMSE, but future demographic, economic, and policy changes are inherently uncertain. Assumptions about price reductions, though plausible, remain idealized.

## 5. Conclusions

In summary, our findings demonstrate that Hib vaccine introduction is a highly cost-effective intervention with dual health and economic benefits, but persistent inequities in vaccine access and disease burden must be addressed to achieve global health equity. These results provide robust empirical support for optimizing national and global vaccine strategies, advancing vaccine equity, and guiding resource allocation decisions. Policymakers, particularly in countries yet to introduce Hib vaccine, should consider these new data in decision-making to maximize both health and economic returns.

## Figures and Tables

**Table 1:** Average years since Hib vaccine introduction and health benefits by subgroup.

Groups	Average Introduce Years	50% Inclusion	Health Benefits of Disease Burden Averted (95% CI)			
			Deaths, Thousand	Death Rate	DALYs, Million	DALY Rate, Thousand
Region						
Africa	15.90	2008	351.77 (8.99–749.33)	954.52 (699.20–1233.56)	24.35 (1.10–53.05)	78.00 (55.13–105.70)
America	20.91	2000	325.97 (8.94–667.68)	366.61 (318.25–496.74)	22.19 (0.99–48.2)	17.61 (17.21–25.28)
Asia	16.19	2009	234.28 (10.31–478.38)	320.21 (244.36–427.61)	16.54 (0.56–36.26)	18.29 (15.38–26.45)
Europe	23.00	1994	287.96 (3.34–572.01)	306.84 (216.89–463.70)	19.66 (0.61–41.59)	7.72 (10.34–12.81)
Oceania	17.14	2005	121.14 (0.45–255.91)	178.54 (149.44–235.35)	8.22 (0.31–17.99)	12.45 (9.45–18.38)
Income						
Low income	15.91	2008	542.07 (9.97–1092.96)	584.44 (438.19–853.09)	36.96 (1.2–78.72)	18.62 (20.95–29.18)
Lower-middle income	16.82	2008	174.90 (2.97–382.35)	559.20 (398.89–711.62)	12.10 (0.22–26.88)	47.6 (32.61–64.35)
Upper-middle income	16.83	2003	300.02 (12.51–626.89)	603.75 (473.36–789.94)	20.88 (1.38–45.82)	46.79 (35.43–64.92)
High income	21.75	1998	304.14 (6.59–621.11)	379.32 (317.71–502.32)	21.03 (0.77–45.68)	21.07 (18.53–30.16)
Gavi						
Non-Gavi	18.98	2002	978.19 (23.33–1981.71)	1160.54 (920.15–1615.50)	67.14 (2.47–144.58)	52.73 (50.32–78.48)
Gavi	16.69	2007	342.93 (8.71–741.6)	966.17 (708.00–1241.46)	23.83 (1.10–52.52)	81.35 (57.19–110.14)

## Data Availability

Data are available in public, open access repositories.

## Supplementary Materials

The following supporting information can be downloaded at: https://www.mdpi.com/article/10.3390/vaccines13121197/s1.
